# DNA Double-Strand Breaks Induced in Human Cells by 6 Current Pesticides: Intercomparisons and Influence of the ATM Protein

**DOI:** 10.3390/biom12020250

**Published:** 2022-02-03

**Authors:** Laurène Sonzogni, Mélanie L. Ferlazzo, Adeline Granzotto, Béatrice Fervers, Laurent Charlet, Nicolas Foray

**Affiliations:** 1 INSERM U1296 Unit “Radiation: Defense, Health, Environment”, Centre Léon-Bérard, 69008 Lyon, France; laurene.sonzogni@inserm.fr (L.S.); melanie.ferlazzo@inserm.fr (M.L.F.); adeline.granzotto@inserm.fr (A.G.); beatrice.fervers@lyon.unicancer.fr (B.F.); 2 Cancer & Environment Department, Centre Léon-Bérard, 69008 Lyon, France; 3 ISTerre Team, University Grenoble Alpes, 38000 Grenoble, France; charlet38@gmail.com

**Keywords:** pesticides, toxicity, DNA double-strand breaks, ATM, immunofluorescence

## Abstract

A mechanistic model from radiobiology has emerged by pointing out that the radiation-induced nucleo-shuttling of the ATM protein (RIANS) initiates the recognition, the repair of DNA double-strand breaks (DSB), and the final response to genotoxic stress. More recently, we provided evidence in this journal that the RIANS model is also relevant for exposure to metal ions. To document the role of the ATM-dependent DSB repair and signaling after pesticide exposure, we applied six current pesticides of domestic and environmental interest (lindane, atrazine, glyphosate, permethrin, pentachlorophenol and thiabendazole) to human skin fibroblast and brain cells. Our findings suggest that each pesticide tested may induce DSB at a rate that depends on the pesticide concentration and the RIANS status of cells. At specific concentration ranges, the nucleo-shuttling of ATM can be delayed, which impairs DSB recognition and repair, and contributes to toxicity. Interestingly, the combination of copper sulfate and thiabendazole or glyphosate was found to have additive or supra-additive effects on DSB recognition and/or repair. A general mechanistic model of the biological response to metal and/or pesticide is proposed to define quantitative endpoints for toxicity.

## 1. Introduction

While pesticides are abundantly used in agriculture, there is increasing evidence that their contact and handling may be associated with various cancer, neurodegenerative, respiratory, metabolic, and developmental diseases [1,2,3,4,5,6,7]. However, the complexity of the molecular mechanisms of toxicity and carcinogenesis, the lack of specificity of some biomarkers, the diversity of cellular models, the number of experimental protocols applied, and the absence of data intercomparisons have made the quantitative evaluation of the risks linked to pesticides difficult [6,8,9].

Pesticides represent an actual issue of public health through two essential clinical features, at least: toxicity and cancer risk [6]. By considering ionizing radiation (IR) or chemical agents other than pesticides, there is a very documented causal and quantified link between toxicity and unrepaired DNA double-strand breaks (DSB), on one hand, and genomic instability, cancer, and misrepaired DSB, on the other [10,11]. However, unlike IR and some specific chemotherapeutic drugs, the oxidative stress produced in cells by a great majority of chemical drugs may be too energetically low to generate DSB directly and/or current environmental or occupational exposure do not involve sufficiently high concentrations of pesticides to permit a robust measurability and/or the detection of any clinical effect. This situation appears to be similar to the questions raised by low doses of IR [11,12,13,14]. Conversely, indirect DSB, notably produced by numerous DNA single-strand breaks (SSB) generated from biochemical reactions and/or uncontrolled genome maintenance linked to individual susceptibility, may contribute to the early steps of toxicity and/or cancer, suggesting that the individual factor may add some discontinuities to the IR dose–drug concentration–effect curves [11,13,15,16,17]. Furthermore, the affinity of some chemicals with some others also raises the question of the additivity or supra-additivity of the effects due to combined drugs, which adds extra complexity to the quantitative evaluation of the risks. This is notably the case of metals and pesticides, for which cocktail effects have already been documented [18,19].

The non-homologous end-joining (NHEJ) pathway is the predominant DSB recognition and repair pathway in human cells [11,20]. Notably, the DSB sites, whatever their origin, are recognized very early after the induction of stress by the phosphorylation of the variant H2AX histone proteins (γH2AX), which requires the normal activity of the ATM kinase in the nucleus [21]. The ATM protein is a major actor of the individual response to DNA-breaking agents. To date, there is very well documented evidence that the radiation-induced nucleo-shuttling of the ATM protein (RIANS) permits a robust prediction of radiosensitivity and cellular toxicity [22,23,24,25,26,27,28]. The RIANS model is based on the following molecular steps of the stress response: (1) oxidative stress results in both the induction of DSB in the nucleus and the monomerization of the ATM dimers which are mainly localized in cytoplasm; (2) the resulting ATM monomers shuttle from the cytoplasm to the nucleus; (3) the ATM monomers phosphorylate H2AX histones which produce nuclear γH2AX foci at the DSB sites (easily visible by immunofluorescence), and contribute to the recognition of DSB, repaired by the NHEJ pathway; (4) complete DSB repair produces the trans-autophosphorylation of ATM proteins (pATM), which form ATM dimers in the nucleus and nuclear pATM foci at the DSB sites, again easily visible by immunofluorescence [22,27,28,29]. A delay in the ATM nucleo-shuttling may be caused by an overproduction of some ATM phosphorylation substrate proteins (called X-proteins) in cytoplasm, which sequestrate the ATM monomers. Consequently, either the unrecognized DSB remain unrepaired and participate in cell death and toxicity, or else they are misrepaired by error-prone recombination-like pathways and participate in cell transformation and cancer [22,27,28,29]. While it has been abundantly documented in a response to IR, the RIANS model has been recently validated in this journal for the exposure of human cells to 12 metallic species [13].

Here, we examined whether a model based on the nucleo-shuttling of the ATM protein is also relevant for the exposure to pesticide. To this aim, human skin fibroblast and brain astrocyte cells from different RIANS statuses were exposed to six current pesticides of domestic and environmental interest, namely gamma-hexachlorocyclohexane (lindane, HCH) [30,31], atrazine (ATR) [32], glyphosate (GBH) [33], permethrin (PER) [34], pentachlorophenol (PCP) [35,36], and thiabendazole (TBZ) [37] (Table 1), and subjected to immunofluorescence, with the RIANS biomarkers as endpoints. The general objective of this paper is to better identify, document, and quantify the molecular steps of the individual response to pesticides that may lead to cellular death, whatever its form. All the biomarkers applied were therefore deliberately chosen upstream of apoptosis or necrosis.

## 2. Materials and Methods

### 2.1. Cell Lines 

Two types of human cells were used in this study: skin fibroblasts and brain astrocytes. All the cell lines tested were untransformed to avoid any bias linked to genomic stability. Furthermore, all the experiments were performed with cells in the plateau phase of growth (95–99% in G0/G1) to overcome any cell cycle effects, and to focus on the potential perturbations of the NHEJ pathway, the DSB repair and signaling pathway the most predominant in humans, which may be caused by exposure to pesticides. 

Skin fibroblasts were routinely cultured at 37 °C in 5% CO_2_ humid conditions as monolayers with Dulbecco’s modified Eagle’s minimum medium (DMEM) (Gibco-Invitrogen-France, Cergy-Pontoise, France), supplemented with 20% fetal calf serum, penicillin, and streptomycin. The origin and the radiobiological features of the RIANS-normal radioresistant 1BR3, 149BR controls were published elsewhere [38,39]. It is noteworthy that cellular radioresistance was generally defined by a clonogenic cell survival fraction at 2 Gy higher than 50% [11,38,39]. The radiosensitive RIANS-delayed 08HNG fibroblasts were provided from a skin biopsy from a donor who showed adverse tissue reaction after anti-cancer radiotherapy. The 08HNG cell line belongs to the “COPERNIC” collection managed by our lab and approved by the regional Ethical Committee. Cell lines were declared under the numbers DC2008-585, DC2011-1437, and DC2021-3957 to the Ministry of Research. The radiobiological database was protected under the reference as IDDN.FR.001.510017.000.D.P.2014.000.10300 [22].

The human cortex (Ha; #1800), hippocampus (Hah; #1830), and spinal cord (Hasp; #1820) astrocyte cells were purchased from ScienCell Research Laboratories (Carlsbad, CA, USA) and routinely cultured as monolayers at 37 °C in 5% CO_2_ humid conditions, with a specific culture medium provided by the same manufacturer (#1801) supplemented with 20% fetal bovine serum (#0010), penicillin/streptomycin solution (#0503), and growth supplement (#1852). The radiobiological features of these brain astrocytes were published elsewhere [40].

### 2.2. Pesticides

All the pesticides tested here were purchased from Sigma-Aldrich France, Saint-Quentin-Fallavier, France): gamma-hexachlorocyclohexane (lindane, HCH; #49049), atrazin (ATR; #45330), glyphosate (GBH; #45521), permethrin (PER; #45614), pentachlorophenol (PCP; 48692), and thiabendazole (TBZ; #45684) (Table 1). Pesticides were diluted and were added directly to the culture medium. A range of pesticide concentrations covering 0.01 µM to 1000 µM was systematically applied for 2 reasons: (1) to investigate the largest spectrum of exposures at which molecular events are measurable; (2) to analyze data with mathematical functions in a significant log scale.

### 2.3. Metals

The CuCl_2_ and CuSO_4_ metal species were purchased from Sigma-Aldrich (#751944 and #451657), respectively. Metals were diluted into culture medium for 24 h at the indicated concentrations. The 99% purified metal-pesticides complexes solution was prepared according to a protocol detailed elsewhere [41].

### 2.4. X-Rays Irradiations

Irradiations were performed with a 6 MeV X-rays medical SL 15 irradiator (Philips, Amsterdam, The Netherlands) (dose-rate: 6 Gy.min^−1^) at the Anti-Cancer Centre Léon-Bérard (Lyon, France) [22,39]. In all the experiments, a dose of 2 Gy was applied. It is noteworthy that 2 Gy X-rays represented a reference dose equivalent to a session of standard anti-cancer radiotherapy.

### 2.5. Immunofluorescence

The immunofluorescence protocol was described elsewhere [42,43]. Briefly, cells were fixed in paraformaldehyde for 10 min at room temperature, and were permeabilized in 0.5% Triton X-100 solution for 5 min at 4 °C. Primary and secondary antibody incubations were performed for 40 and 20 min at 37 °C, respectively. The anti-*γH2AX^ser139^* antibody (#05636; Upstate Biotechnology-Euromedex, Mundolsheim, France) was used at 1:800. The monoclonal anti-mouse anti-*pATM^ser1981^* (#ab2888) from Abcam (Cambridge, UK) was used at 1:100. Incubations with anti-mouse fluorescein (FITC) and rhodamine (TRITC) secondary antibodies were performed at 1:100 at 37 °C for 20 min. Slides were mounted in 4′,6′ Diamidino-2-Phenyl-indole (DAPI)-stained Vectashield (Abcys, Paris, France) for scoring micronuclei and mitoses, and examined with an Olympus fluorescence microscope. DAPI staining also indirectly permitted the evaluation of the yield of G_1_ cells (nuclei with homogeneous DAPI staining), S cells (nuclei showing numerous γH2AX foci), G_2_ cells (nuclei with heterogeneous DAPI staining), and metaphase (visible chromosomes). 

The foci scoring procedure applied here has received the certification agreement of CE mark and ISO-13485 quality management system norms. Our foci scoring procedure also developed some features that are protected in the frame of the Soleau Envelop and patents (FR3017625 A1, FR3045071 A1, EP3108252 A1) [43]. More than 50 nuclei were analyzed per experiment, and at least 3 independent replicates were performed for each condition [22].

### 2.6. Statistical Analysis

The response curves data were fitted to the sigmoidal function, as defined in Table 2. The γH2AX and pATM foci kinetic data were fitted to the Bodgi’s formula, which described the kinetics of appearance/disappearance of nuclear foci formed by some protein relocalizing after genotoxic stress [44]. The inhibition of the DSB recognition was defined as 100 × (1 − Np+/Np−), in which Np+ and Np− are the numbers of γH2AX foci assessed 10 min post-irradiation with and without pesticide, respectively. Each quantitative correlation between series of two corresponding data values was characterized by a mathematical formula and its correlation coefficient. The Wilcoxon signed rank test was not applied to the different hierarchies established between pesticides, since the minimal sample size required was lower than 16 samples. Statistical significance between data points was verified with the one-way ANOVA test. Statistical analysis was performed by using Kaleidagraph v4 (Synergy Software, Reading, PA, USA).

## 3. Results

### 3.1. Some Pesticides May Induce Persistent DSB in Human Fibroblasts

**Figure 1:**
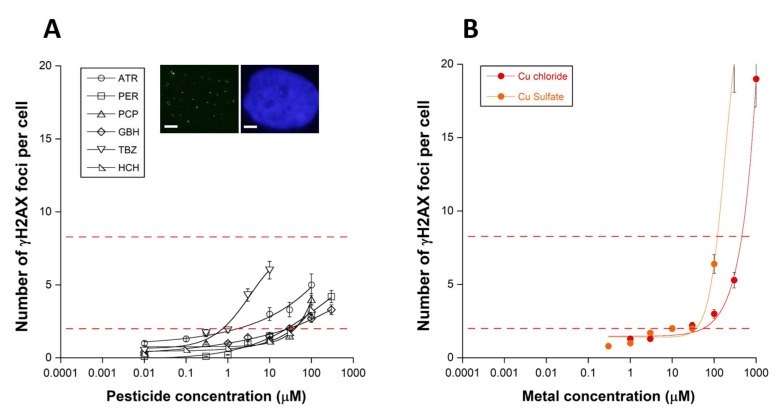

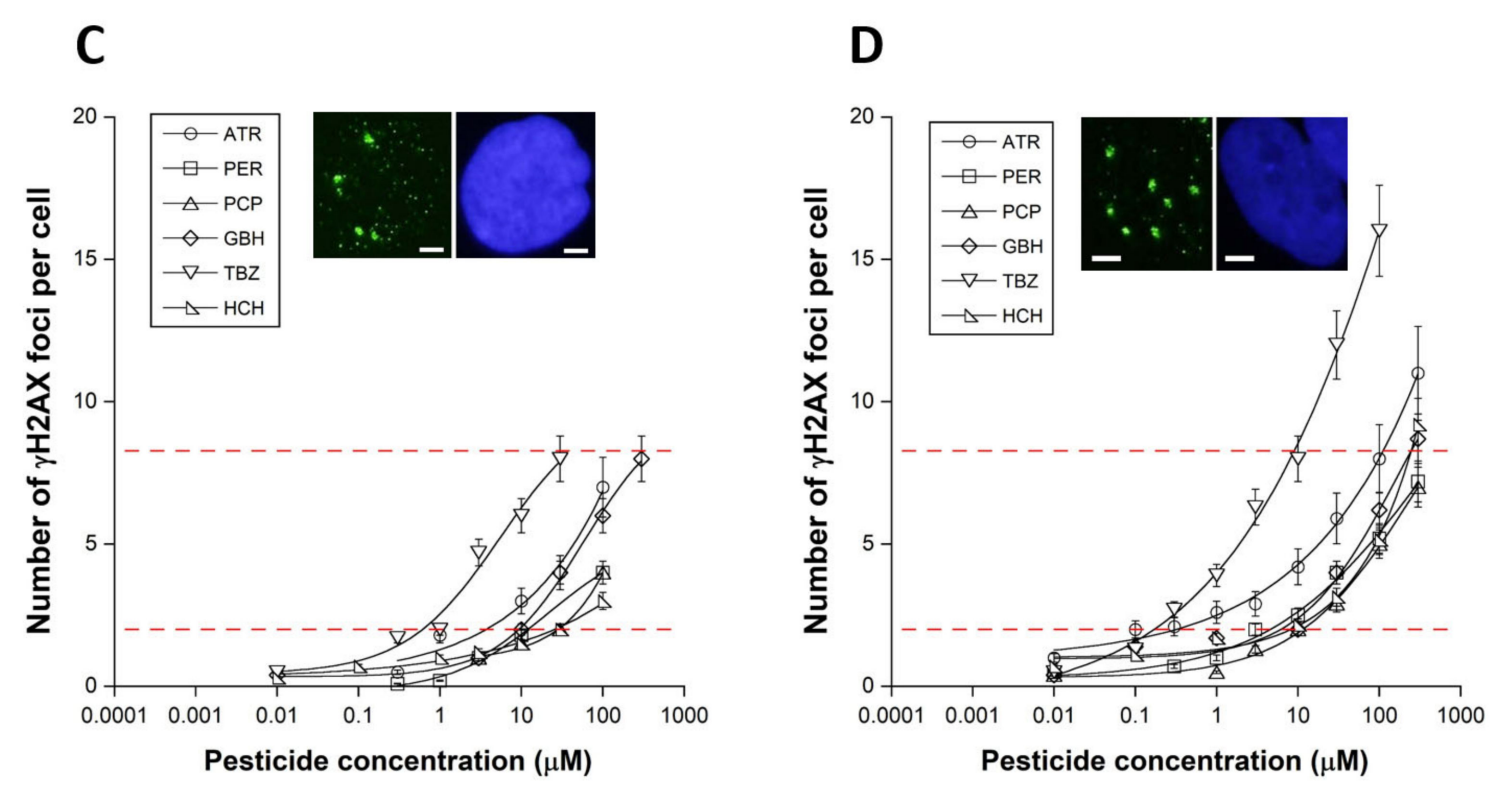
Unrepaired DSB after exposure to pesticides and metal. Number of γH2AX foci per cell in the human untransformed radioresistant 1BR3 cells after incubation for 24 h with the indicated pesticide (**A**) or with CuSO_4_ and CuCl_2_ solutions (**B**). Number of γH2AX foci per cell in the human untransformed radioresistant 149BR (**C**) and delayed-RIANS 8HNG (**D**) cells after incubation for 24 h with the indicated pesticide. Each plot represents the mean ± standard error (SEM) of four replicates. Dotted lines represent the induction of the induction of two or eight γH2AX foci per cell Inserts: Representative examples of γH2AX and DAPI-counterstained images obtained with 1 µM PER (**A**), 3 µM TBZ (**C**) and 100 µM ATR (**D**). White bar represents 5 µm.

The number of the nuclear γH2AX foci was assessed in the human radioresistant and RIANS-normal 1BR3 fibroblasts 24 h after the introduction of pesticide molecules in the culture medium. The exposure of the 1BR3 cells to pesticides resulted in the appearance of γH2AX foci, suggesting the induction of DSB managed by the NHEJ pathway. All the response curves obeyed the similar sigmoidal functions of the pesticide concentration, but the rate of DSB production appeared to be specific to each pesticide tested (Figure 1A; Table 2). Three specific pesticide concentration ranges have been identified: (1) one range in which the number of γH2AX foci was not significant and did not increase significantly with the pesticide concentration; (2) one range in which the number of γH2AX foci was significant and increased, generally linearly, with the pesticide concentration; (3) one range in which the number of γH2AX foci increased exponentially. It is noteworthy that, in our conditions, no plateau was reached with the highest concentrations tested.

Similar γH2AX foci sigmoidal curves have been observed after exposure to metals [13]. A representative example is shown in Figure 1B with the exposure of 1BR3 cells to copper (Cu), sulfate (CuSO_4_), or chloride (CuCl_2_) (Figure 1B). Similar γH2AX foci curves were also obtained with another human fibroblast (149BR) cell line that derived from an apparently healthy donor, and shows normal RIANS (Figure 1C). Conversely, the 08HNG fibroblasts that show delayed RIANS elicited significantly more γH2AX foci than both 1BR3 and 149BR cell lines from 3 µM pesticides for TBZ (*p* < 0.01) and ATR (*p* < 0.05), suggesting that the RIANS status may condition the rate of the production of DSB induced in a specific pesticide concentration-dependent manner (Figure 1D).

In our hands, the average background level of untransformed human fibroblasts was found to be lower than 2 γH2AX foci per cell [22,29]. Furthermore, below this threshold, no significant clinical feature has been observed in donors, while a higher number of γH2AX foci per cell can reveal genomic instability and toxicity. Lastly, it is noteworthy that more than eight γH2AX foci per cell were shown to correspond to hyper-radiosensitivity and an extreme toxicity [10]. No pesticide concentration tested in this study reached the threshold of eight γH2AX foci per cell, to the notable exception of TBZ and ATR. 

The threshold drug concentration to reach more than two γH2AX foci per cell, called TMC_>2_, was deduced from data and analyzed. Each pesticide tested was found to be characterized by a specific TMC_>2_ value of some µM, reflecting the capacity of the pesticide to induce DSB (Table 2). By taking the Cu metallic species data published recently as references [13], TBZ and ATR were found to induce more DSB than CuCl_2_ and CuSO_4_, while the other pesticides tested were found to induce less DSB. 

By plotting TMC_>2_ data values obtained from the RIANS-delayed 08HNG fibroblast cell lines against those obtained from RIANS-normal ones (namely 1BR3 and 149BR), a quantitative correlation appeared (y = 0.238x − 0.232; r = 0.985), revealing that the TMC_>2_ from the RIANS-delayed 08HNG cells were 4.2 (=1/0.238) times higher than those of the RIANS-normal control cells without any change in the rank order of pesticides (Figure S1). Altogether, our findings suggest that the pesticides tested induce persistent/slowly repairable DSB at specific rate that depends on the pesticide nature and the RIANS status.

### 3.2. Some Pesticides may Induce Persistent Micronuclei in Human Fibroblasts

Micronuclei are considered as the cytogenetic consequence of the unrepaired DSB propagated to the mitotic phase [45]. Micronuclei were assessed directly in the same microscopic slides as those used for the γH2AX foci study: no cytokinesis block of cytochalasin B was applied, as generally performed in the micronuclei assays, even if the binucleated micronuclei notion was preserved. However, the space between two nuclei with micronuclei may appear large, due to the experimental protocol of immunofluorescence applied to cells seeded in glass slides [46]. Such a protocol was applied to permit direct data intercomparison with γH2AX foci, and to avoid any bias linked to the cytokinesis block. This protocol permits one to compare data obtained from exposure to other DNA breaking agents, such as IR and metals [13,22].

**Figure 2:**
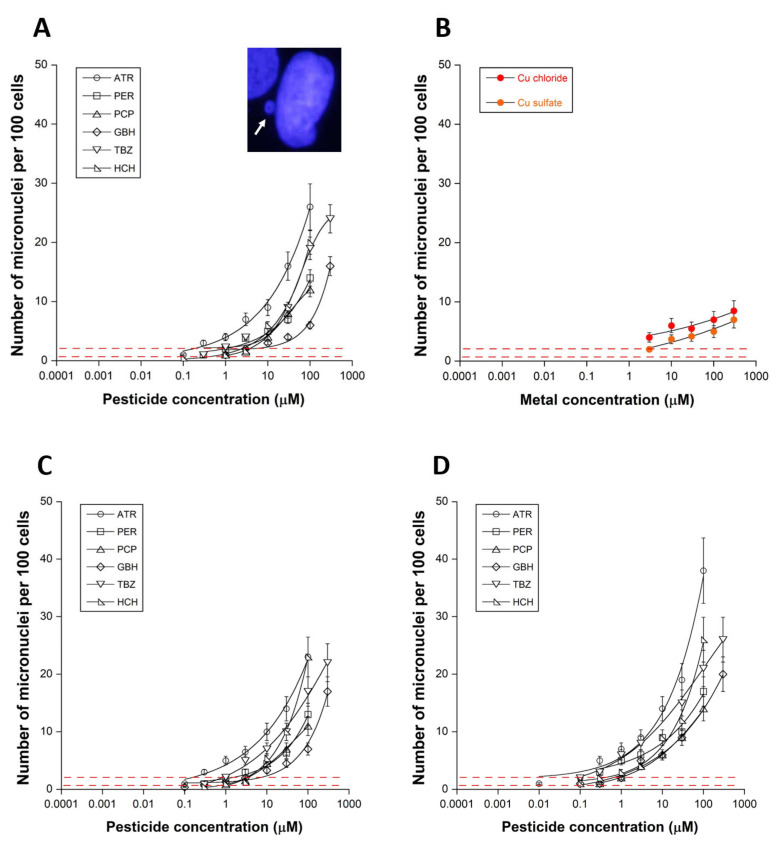
Residual micronuclei after exposure to pesticides and metals. Number of micronuclei per 100 cells in the human untransformed RIANS-normal radioresistant 1BR3 cells after incubation for 24 h with the indicated concentration of pesticides (**A**) or with CuSO_4_ and CuCl_2_ solutions (**B**). Number of micronuclei per 100 cells in the human untransformed RIANS-normal radioresistant 149BR (**C**) and RIANS-delayed 08HNG (**D**) cells after incubation for 24 h with the indicated concentration of pesticides. Each plot represents the mean ± standard error (SEM) of four replicates. Insert: Representative examples of γH2AX and DAPI-counterstained images obtained at 1 µM TBZ. White bar represents 5 µm.

The number of micronuclei induced by the pesticides tested also obeyed a pseudo-sigmoidal function of the pesticide concentration similar to those observed with the γH2AX data (Figure 1A,C,D and Figure 2A,C,D). It is noteworthy that CuCl_2_ and CuSO_4_ also induce similar curves when micronuclei are used as an endpoint (Figure 1B and Figure 2B). 

**Figure 3:**
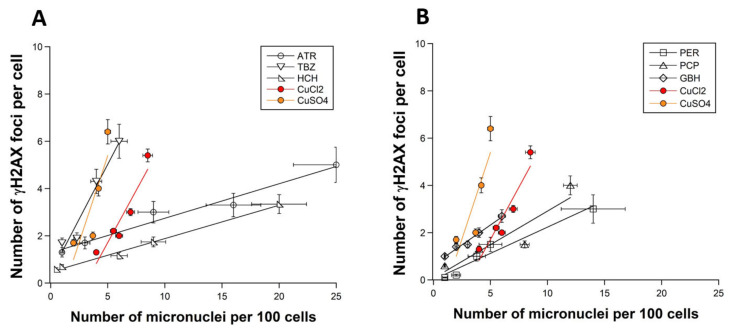
Relationships between the residual γH2AX and micronuclei data. Plots correspond to ATR, TBZ and HCH (**A**), and PER, PCP, and GBH data (**B**), respectively. Cu data have been reproduced in the two panels. The means ± standard error (SEM) of γH2AX data shown in Figure 1 was plotted against the corresponding means ± SEM of micronuclei data shown in Figure 2. Linear function was used to fit data.

By plotting the γH2AX data against the corresponding micronuclei data, a quantitative link appeared between the two endpoints, consistently with the well-documented link between micronuclei and unrepaired DSB: the higher the number of residual γH2AX foci, the higher the yield of residual micronuclei (Figure 3A,B) protocol [13,22]. Here, the number of γH2AX foci was expressed per cell, while the number of micronuclei was expressed per 100 cells, since micronuclei formation is less frequent than DSB production. TBZ was associated with the highest γH2AX foci/micronuclei ratio, suggesting that about 1 γH2AX foci produced by TBZ can lead to 1 micronucleus per 100 cells. The corresponding ratios for the other pesticides tested were found to be much lower: (GBH: 0.336; PCP: 0.284; PER: 0.222; ATR: 0.145; HCH: 0.141) (Figure 3A,B). It is noteworthy that the corresponding γH2AX foci/micronuclei ratios for CuCl_2_ and CuSO_4_ were found to be 0.886 and 1.46 [13], respectively, i.e., close to the ratio obtained with TBZ. These last findings suggest that TBZ and Cu salts share similar properties to propagate the unrepaired DSB, resulting in micronuclei formation. Regarding the other pesticides, we are reminded that some DSB unrecognized by the NHEJ pathway (i.e., not labelled by γH2AX foci) can generate micronuclei through impaired pathways different from NHEJ. 

By plotting TMC_>2_ values obtained from γH2AX data against the corresponding values obtained from micronuclei data (TMC for reaching 2 micronuclei per 100 cells), a quantitative correlation appeared, suggesting that, whatever the pesticide tested, the higher the capacity to induce DSB managed by NHEJ, the higher the yield of micronuclei (Figure S2). Similar conclusions were reached with the other cell lines tested (data not shown).

### 3.3. Influence of the Presence of Pesticide During the RI DSB Recognition and Repair Process

The data described above showed that pesticides induce a low number of DSB at concentrations lower than 10 µM. Hence, the data described above did not permit to verify statistically whether pesticide molecules inhibit the recognition and/or repair of the DSB that they contribute to induce. Hence, to better understand the influence of pesticides on the DSB recognition and repair steps, we used X-rays, a physical agent that induces a very well-documented number of DSB per cell per given dose, without any chemical interaction with pesticides [47,48]. A DSB induction rate of 37 ± 4 DSB per Gy per cell has been generally found in human fibroblasts, whatever their radiosensitivity and RIANS status [11]: at a dose of 2 Gy applied for 2 min, the number of DSB induced by IR was much higher than the DSB induced by pesticides. 

**Figure 4:**
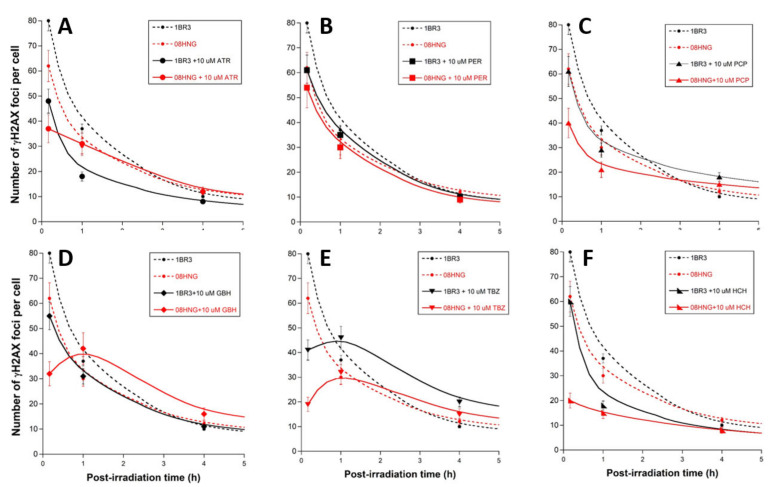
γH2AX kinetics after exposure to pesticide and X-rays. Number of γH2AX foci as a function of post-irradiation time in the human untransformed radio-resistant RIANS-normal 1BR3 (black plots) and RIANS-delayed 08HNG (red plots) fibroblasts incubated for 24 h, with the indicated concentrations of pesticides (solid line) or not (dotted line), and irradiated (2 Gy X-rays) thereafter ((**A**): ATR; (**B**): PER; (**C**): PCP; (**D**): GBH; (**E**): TBZ; (**F**): HCH). Each plot represents the mean ± standard error (SEM) of 3 replicates. It is noteworthy that the 24 h data were not found to be dependent of the presence of pesticides before irradiation (data not shown).

Cells were exposed to pesticides for 24 h, then exposed to 2 Gy X-rays. After culture medium renewal immediately after irradiation, a repair time ranging from 10 min to 24 h was applied (Figure 4). The γH2AX foci kinetics obtained with pesticides were not found to be different from those obtained without pre-exposure to pesticides, with the notable exception of the numbers of γH2AX foci assessed 10 min post-irradiation (Figure 4). Indeed, the pre-exposure to pesticide molecules systematically resulted in significantly decreasing the number of early γH2AX foci (*p* < 0.001 for all the pesticides), suggesting that their presence during irradiation influences DSB recognition. Conversely, the number of γH2AX foci assessed 24 h post-irradiation was not found to be significantly influenced by the presence of pesticides (*p* > 0.1 for all the pesticides), suggesting that the repair of recognized DSB was not affected by the presence of pesticides (data not shown).

**Figure 5:**
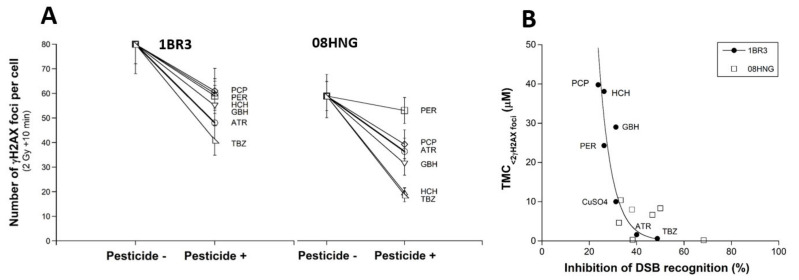
Power inhibition of DSB recognition of the pesticides tested. (**A**) The γH2AX foci assessed 10 min post-irradiation shown in Figure 4 in the RIANS-normal (1BR3) and the RIANS-delayed 08HNG fibroblasts, with or without pre-exposure to pesticides (pesticide+ and pesticide-, respectively). Each plot represents the mean ± standard error (SEM) of three replicates. These data helped us to calculate the inhibition power of DSB recognition (see Materials and Methods). (**B**) The TMC_>2γH2AX foci_ values deduced in the above chapters were plotted against the corresponding inhibition power of the DSB recognition for both 1BR3 and 08HNG cells. The 1BR3 data were fitted to the following formula: y = 3661.4 exp(−0.1815x) (r = 0.87). The CuSO_4_ data were taken from [13].

In order to investigate the influence of each pesticide tested on the radiation-induced DSB recognition according to the RIANS status, the number of γH2AX foci assessed 10 min post-irradiation in the 1BR3 and 08HNG cells, with or without pesticide, was plotted together (Figure 5A). A new rank order reflecting the power of DSB recognition inhibition for each pesticide, from the highest to the weakest, was obtained: TBZ > HCH > GBH > ATR > PCP > PER. This rank order may change with the RIANS status of the cell lines tested, but TBZ systematically elicited the strongest inhibition of DSB recognition, and PER and PCP elicited the weakest inhibition of DSB recognition (Figure 5A). To investigate the meaning of these findings further, we quantified the inhibition power of DSB recognition, due to the presence of pesticide from the γH2AX foci data assessed for 10 min (see Materials and Methods). This new parameter was plotted against the corresponding TMC_>2_ values: a quantitative correlation appeared for the RIANS-normal 1BR3 cells, suggesting that the TMC_>2_ values the lower, and the inhibition power of DSB recognition the stronger (Figure 5B). In other words, the less that the DSB are recognized by NHEJ, the greater the DSB induction power, which is consistent with the hypothesis that DSB should be recognized to be repaired. It is noteworthy that the fitting formula was an exponential function with an infinite TMC_>2_ corresponding to a lack of DSB recognition, but the data shown in Figure 5B may also suggest a sigmoidal function. Interestingly, such a correlation did not appear with the RIANS-delayed 08HNG cells, likely because the inhibition of the DSB recognition is much stronger in this cell line, and the TMC_>2_ values of the pesticides tested belong to a more limited range of concentration. 

### 3.4. Influence of the Presence of Pesticide on the Nucleo-Shuttling of the ATM Protein

**Figure 6:**
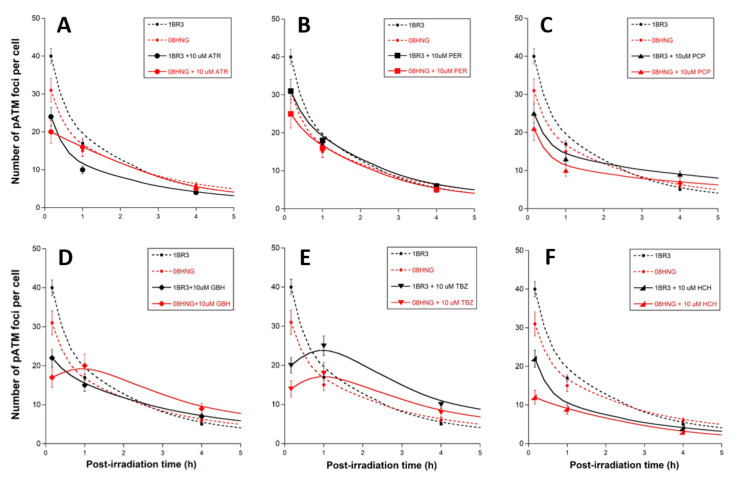
pATM foci kinetics after exposure to pesticide and X-rays. Number of γH2AX foci as a function of post-irradiation time in the human untransformed radioresistant RIANS-normal 1BR3 (black plots) and RIANS-delayed 08HNG (red plots) fibroblasts incubated for 24 h with the indicated concentrations of pesticides (solid line) or not (dotted line) and irradiated (2 Gy X-rays) thereafter ((**A**): ATR; (**B**): PER; (**C**): PCP; (**D**): GBH; (**E**): TBZ; (**F**): HCH). Each plot represents the mean ± standard error (SEM) of three replicates. It is noteworthy that the 24 h data were not found to be dependent on the presence of pesticides before irradiation.

The data described above suggested that the presence of the pesticides tested may inhibit the DSB recognition via the ATM-dependent NHEJ pathway (conditioning the RIANS status). We examined, therefore, the nuclear relocalization of the auto-phosphorylated form of the ATM protein, reflecting its kinase activity in the nucleus [29]. By applying *anti-pATM* immunofluorescence to cells exposed to pesticide and 2 Gy X-rays, in the same conditions as described above, the numbers of the nuclear pATM foci assessed 10 min after irradiation were assessed (Figure 6).

Similarly to the γH2AX foci data, the pAΤΜ foci kinetics obtained with pesticides were not found to be different from those obtained without pre-exposure to pesticides, with the notable exception of the numbers of pAΤΜ foci assessed 10 min post-irradiation (Figure 6). The pre-exposure to pesticide molecules systematically resulted in a significantly decreasing number of early pAΤΜ foci (*p* < 0.001 for all the pesticides), suggesting that their presence during irradiation influences the DSB recognition. This last conclusion was consolidated by the quantitative correlation between γH2AX and pATM foci data (Figure S3), and in quantitative agreement with previous studies [26]. The rank order of pesticides that reflects the inhibition power of DSB recognition inhibition deduced from the pATM data was found to be similar to that deduced from γH2AX data (Figure 6 and S3).

### 3.5. Supra-Additive Effect Produced by the Exposure to Combined Copper and Pesticide

All the above data suggested that the presence of TBZ is responsible for a significant production of DSB, and may influence DSB recognition. Furthermore, GBH still raises an important public health and societal problem. In parallel, since CuSO_4_ is widely used as a fungicide, algaecide, and herbicide, TBZ, GBH and CuSO_4_ can be applied together in agriculture, raising the question of the effect of cocktail solutions combining metals and pesticides. We therefore examined the effect of the exposure of the combination of CuSO_4_ and TBZ, on one hand, and CuSO_4_ and GBH, on the other hand, in the same irradiated human fibroblast cell lines used in the experiments described above. In this study, we applied the same molarity for both metal and pesticide i.e.,10 µM for the TBZ + 10 µM CuSO_4_ and 10 µM GBH + 10 µM CuSO_4_. As a first step, the metal and pesticide were added together in the culture medium. 

**Figure 7:**
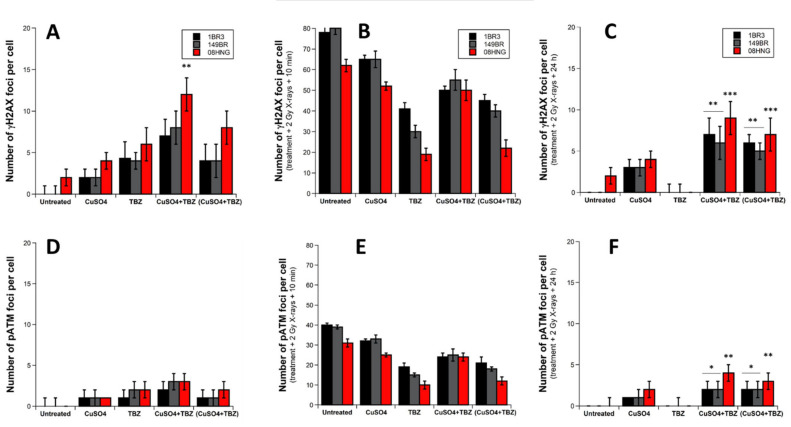
γH2AX and pATM foci from fibroblasts exposed to CuSO_4_ and/or TBZ. Indicated cells were exposed to 24 h incubation with the indicated metal, pesticide, CuSO_4_+TBZ or 99% purified (CuSO_4_+TBZ) complexes solutions, then irradiated at two Gy X-rays and incubated up to 24 h for repair. “Untreated” mention means that neither pesticide nor metal were applied to cells before irradiation. Number of γH2AX and pATM foci per cell assessed before irradiation (**A**,**D**), at 10 min post-irradiation (**B**,**E**) and at 24 h post-irradiation (**C**,**F**), respectively. Each plot represents the mean ± standard error (SEM) of 3 replicates. One, two, or three asterisks represent significant differences from TBZ data with *p* < 0.05, *p* < 0.01, and *p* < 0.001, respectively.

A simultaneous exposure to CuSO_4_ and TBZ for 24 h resulted in more γH2AX foci per cell than metal or pesticide applied separately. This trend was found to be significant for the 08HNG cells only, suggesting that the metal+pesticide combinations tested may produce an additive effect; at least, in the RIANS-delayed cells (Figure 7A). 

Since the numbers of γH2AX foci remained low, we applied the same protocol using 2 Gy X-rays irradiation as that described in the above sections. The number of γH2AX foci assessed 10 min after irradiation in untreated cells reflected the delayed RIANS of the 08HNG cells. The presence of TBZ molecules reduced the DSB recognition consistently with the above data. The presence of CuSO_4_ alone appeared to inhibit DSB recognition, but to a lesser extent than TBZ. Interestingly, the concomitant presence of metal and pesticide did not lead to a lower inhibition of DSB recognition than with TBZ alone, but larger than with CuSO_4_ alone (Figure 7B). The number of γH2AX foci assessed at 24 h post-irradiation revealed that the concomitant presence of metals and pesticides significantly inhibits DSB repair much more than CuSO_4_ or TBZ molecules taken separately, suggesting a supra-additive effect for all the cell lines tested (Figure 7C).

While pesticides molecules, and metals were added simultaneously in the culture medium, they may represent a heterogenous mixture of metal+pesticide complexes and free separated metal, and pesticides molecules may act differently in the DSB induction, recognition, and repair steps. In order to examine whether a facilitated complexation of metals and pesticides before adding them to the culture medium would condition these steps, we added a solution containing 99% purified (CuSO_4_ + TBZ) complexes to the culture medium, and applied it for 24 h to the cells before irradiation. Interestingly, the 99% purified (CuSO_4_ + TBZ) complexes resulted in less γH2AX foci induced than CuSO_4_ + TBZ, suggesting that free CuSO_4_ and/or TBZ molecules were responsible for a large contribution of DSB induction (Figure 7A). Conversely, the numbers of γH2AX foci assessed 10 min after irradiation appeared to be similar to those obtained with TBZ molecules alone, suggesting a large contribution of the 99% purified (CuSO_4_ + TBZ) complexes in the inhibition power of the DSB recognition step (Figure 7B). Regarding the residual γH2AX foci assessed 24 h post-irradiation with the solution containing 99% purified (CuSO_4_ + TBZ) complexes, their numbers were not found to be significantly different from those obtained with CuSO_4_ + TBZ, suggesting that the inhibition of the DSB repair process was essentially due to the metal + pesticides complexes. The corresponding pATM data were in quantitative agreement with the γH2AX foci data (Figure 7D–F).

**Figure 8:**
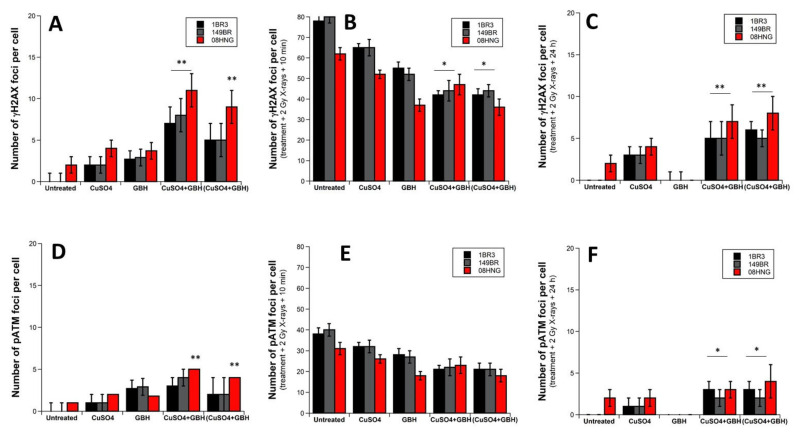
γH2AX and pATM foci from fibroblasts exposed to CuSO_4_ and/or GBH. Indicated cells were exposed to 24 h incubation with the indicated metal, pesticide, CuSO_4_ + GBH or 99% purified (CuSO_4_+GBH) complexes solutions, then irradiated at 2 Gy X-rays and incubated up to 24 h for repair. “Untreated” mention means that neither pesticide nor metal were applied to cells before irradiation. The numbers of γH2AX and pATM foci per cell were assessed before irradiation (**A**,**D**), at 10 min post-irradiation (**B**,**E**), and at 24 h post-irradiation (**C**,**F**), respectively. Each plot represents the mean ± standard error (SEM) of 3 replicates. One or two asterisks represent significant differences from GBH data with *p* < 0.05, *p* < 0.01, respectively.

Similar conditions to those described above were applied for the combination of GBH and CuSO_4_ molecules (Figure 8). While the general conclusions were found to be similar to those reached with the TBZ + CuSO_4_ complexes, it is noteworthy that the inhibition power of the DSB recognition of GBH was found to be lower than that of TBZ. Consequently, the differences between the results obtained from GBH treatment alone and the GBH + CuSO_4_ complexes are less marked. Furthermore, likely because of a very strong affinity of Cu for GBH molecules, the differences between GBH + CuSO_4_ mixtures and 99% (GBH + CuSO_4_) complexes were also less marked (Figure 8). Altogether, these findings suggest that the induction of DSB, the inhibition of DSB recognition, and repair were found to be significant and even higher with the GBH + CuSO_4_ mixtures or purified complexes than with the other conditions tested.

**Figure 9:**
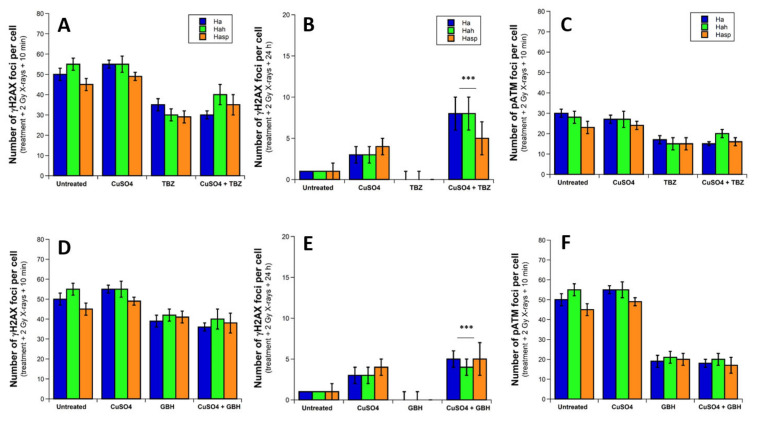
γH2AX and pATM foci from brain astrocytes exposed to CuSO_4_ and/or TBZ or GBH. Indicated cells were exposed to 24 h incubation with the indicated metal, pesticide, metal+pesticide, or 99% purified (metal + pesticide) complexes solutions, then irradiated at 2 Gy X-rays and incubated for up to 24 h for repair. “Untreated” mention means that neither pesticide nor metal were applied to cells before irradiation. The number of γH2AX foci per cell was assessed at 10 min post-irradiation (**A**,**D**) and at 24 h post-irradiation (**B**,**E**), and the number of pATM foci per cell was assessed at 10 min post-irradiation (**C**,**F**) for TBZ and GBH, respectively. Each plot represents the mean ± standard error (SEM) of three replicates. Three asterisks represent significant differences from GBH mean value with *p* < 0.001.

In our previous study about the biological effect of exposure to metal, we have applied the same experimental protocols to skin fibroblasts and a subset of human brain astrocytes from the same donor. Here, the same experimental conditions as described above were applied to human cortex (Ha), hippocampus (Hah), and spinal cord (Hasp) astrocytes (Figure 9).

The concomitant application of metal and pesticide to human brain astrocytes led to a similar inhibition power of DSB recognition than that observed with pesticide alone, but a significantly larger number of unrepaired DSB, suggesting a strong toxicity (Figure 9). No specific differences were observed between brain localizations (human cortex, hippocampus, and spinal cord). Altogether, these findings suggest that (1) human brain astrocytes may show an abnormal response to pesticide leading to toxicity; (2) metal + pesticide cocktail may show an additive- or supra-additive toxic effect to human brain astrocytes.

## 4. Discussion

### 4.1. Limits of the Study and Difficulties to Evaluate the Risks Related to Exposure to Pesticide

There is highly documented evidence that exposure to pesticide causes the significant production of oxidative species, notably like superoxide anions, of which the interactions may result in hydrogen peroxide, a very efficient DNA-breaking agent. This is particularly true for the six pesticides tested here, and especially in human cells [49,50,51,52,53,54]. Hence, exposure to pesticide should result in unrepaired and maybe misrepaired DNA breaks, potentially leading to toxicity and carcinogenicity, respectively. However, as mentioned in Introduction, the causal link between DNA breaks, whether induced directly or indirectly by pesticide molecules and clinical features, has been made difficult by our lack of knowledge of the intrinsic molecular mechanisms, and the diversity of experimental protocols, cellular models, and molecular endpoints applied for a consensual evaluation of the risks (see references in Introduction).

In this study, we have deliberately chosen an experimental approach testing several pesticides rather than a single one, in order to identify and quantify a large spectrum of molecular impairments that will lead to different forms of cellular deaths. Hence, while cellular death and clinical consequences may be strongly dependent on the tissue and the organ exposed to pesticides, our approach deliberately consisted in investigating upstream cellular response to genotoxic stress. Such an approach has already been applied in our recent work on metals [13]. Furthermore, we have chosen to test six current pesticides based on different reports highlighting their interest for public health, notably those found in house dust or those among the most extensively used in agriculture [55,56]. Considering the mechanistic model of individual stress response, the analogy with the individual response to IR [28] or metals [13] and pesticides was obvious, and the RIANS model was therefore naturally chosen as a scientific basis of the present study. Hence, we also did not investigate here the chemical way by which the DNA breaks are induced in presence of pesticides, but we considered the induction, recognition, and repair rates of DSB in the presence of pesticides of which the links with cellular, tissue and clinical features are documented.

The choice of the pesticide concentration range was another limit of this study, since the yield of DNA breaks is generally not measurable for nanomolar concentrations in cells exposed in vitro, while occupational and environmental conditions concern this range. Conversely, the preclinical studies involving rodent models or rabbits allow the application of chronic exposure to pesticide at very low concentrations, but may present some biases vis-à-vis the extrapolation to human cells. Besides, there are few preclinical studies involving many pesticides to allow data intercomparison. Hence, we took particular care when analyzing quantitative correlations between each of the molecular endpoints tested, such as micronuclei and γH2AX and pATM foci (see, notably, Supplementary Data), to propose a mechanistic model that would be coherent mathematically. In future experiments, we have to foresee investigations with chronic exposure. However, the application of low pesticides concentrations for a very long time (several days, several weeks?) may raise practical problems regarding the maintenance of cultured cells for long periods. Furthermore, chronic exposures also raise the question that the induction and repair of DNA damage occur concomitantly, and therefore, mathematical models should be developed to predict, from single exposures data, the behavior of cells exposed chronically. This specific item raises the question of the dependence of the DSB repair rate on the initial dose. These issues are similar to those of the radiobiology of low doses of IR.

Finally, in this study, we have stressed the fact that the response to pesticide exposure may strongly depend on individuals, but also on the nature of the tissue tested: three skin fibroblast cell lines of different RIANS status and three astrocyte cell lines derived from the same donor, but originated from different regions of the brain were tested. To our knowledge, this is the first time that the question of individual and tissue factors is raised with human untransformed skin fibroblasts and brain astrocytes exposed to pesticides. Obviously, further investigations are needed to consolidate our findings with a larger number of other cellular models and molecular endpoints. 

### 4.2. Unrepaired DSB as a Reliable Endpoint to Account for the Toxicity of Pesticides and Predict Cellular Death?

To date, there is highly documented evidence that the number of unrepairable DNA damage and chromosome breaks may serve as a measure of toxicity [57]. In this study, our findings suggest that the presence of pesticides may produce DSB, but to a relatively low extent. Hence, to ask whether the presence of pesticide may alter the DSB recognition and/or repair, a significant and well-characterized number of DSB was necessary to increase the robustness of our analysis. As applied in a previous report, IR appear to be an idealistic DNA-breaking agent, since no residue may alter or interact with the action of pesticide molecules in the DSB repair and signaling processes [13]. 

Regarding the γH2AX and pATM foci data assessed early after the exposure to pesticide, it appeared obvious that the presence of pesticide may influence the ATM-dependent phosphorylation of the H2AX at the DSB sites that conditions DSB recognition and triggers DSB repair by NHEJ. As specified above, a similar situation has already been documented in human radiosensitive cells [28], and in human cells exposed to metals [13]. Through the RIANS model, these findings are consistent with the hypothesis that the ATM monomers formed in cytoplasm by the oxidative stress due to pesticide molecules are delayed in their diffusion to the nucleus. Consequently, the flux of ATM monomers entering the nucleus is significantly reduced, and the number of γH2AX and pATM foci is lower than expected. This hypothesis was strengthened by the data obtained in RIANS-delayed cells, which showed a stronger inhibition of DSB recognition than RIANS–normal ones.

The consequences of a delayed nucleo-shuttling of the ATM monomers may be double: (1) some DSB that are not recognized by the early step of the NHEJ pathway may be unrepaired and participate in the letal effect. These DSB may represent a significant subset of DNA damage that cumulates with the recognized, but unrepairable, DSB, that were revealed by the residual γH2AX foci at 24 h post-stress. Both these DSB subsets may be responsible for micronuclei, cellular death, and toxicity; (2) some DSB that are not recognized by the early step of the NHEJ pathway may be managed by an alternative DSB repair pathway, such as the recombination-like one, and become mis-repaired DSB. These DSB may participate in the carcinogenic process, but further investigations are, however, needed to follow these two categories of DSB after the stress by specific biomarkers that should be independent of the toxic (letal) effect. Particularly in the case of an uncontrolled error-prone recombination-like process, a phenomenon producing a very large number of additional DNA breaks has been observed: this is the hyper-recombination phenomenon [11,58,59,60]. The hyper-recombination phenomenon has been systematically associated with cancer proneness [11,58]. Even at low concentrations of pesticides, a small number of initial DNA breaks may be amplified by the hyper-recombination phenomenon, making the relationship between the biological effect and the pesticide concentration non-linear, as reported elsewhere [13,61]. An analysis of the exacerbated response to the pesticide exposure of fibroblasts from DSB repair-defective donors is required to better understand the specificity of the individual factor. When the TMC_>2_, H2AX/micronuclei ratio and DSB recognition inhibition power are plotted against IARC carcinogenicity group classification, it appears that these parameters cannot predict carcinogenicity, and suggest that biomarkers reflecting cancer proneness are also needed (Figure S4).

### 4.3. Toward a Unified Model for Understanding the Response to Pesticides Combined with Metals?

**Figure 10:**
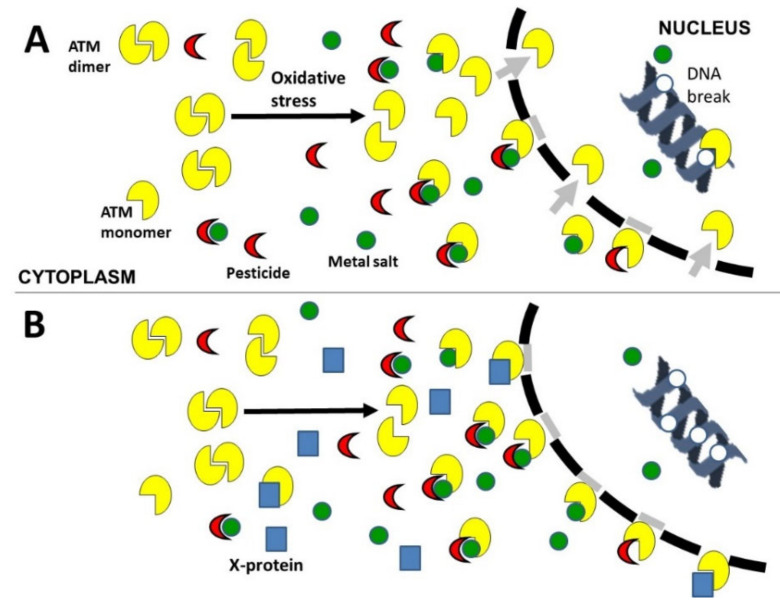
Mechanistic model of metal and pesticide action based on the nucleo-shuttling of the ATM protein. As detailed in discussion, the presence of metal and/or pesticide may produce a significant oxidative stress that contributes to monomerize ATM dimers and induce DSB. Some metal or pesticide molecules or metal+pesticide complexes may bind to ATM monomers and delay their nucleo-shuttling, limiting therefore the DSB recognition by the NHEJ pathway. There are two possible consequences: either DSB are unrepaired, which triggers cell death and toxicity, or DSB are misrepaired by an alternative DSB repair pathway, which triggers genomic instability and carcinogenicity. Panels A and B describe the model in RIANS-normal (**A**) or RIANS-delayed (**B**) cells.

Our findings related to the copper-pesticide complexes reveal that pesticide and copper may be characterized by specific DSB induction, recognition, and repair rates. The affinity of pesticides for copper and TBZ and GBH has been documented abundantly [41,62]. From our findings, it appears that pesticides alone, or as equimolar metal+pesticide complexes, show similar power of DSB recognition inhibition, but stronger than metal alone. Since pesticides are more complex molecules than metallic salts, these data are consistent with the hypothesis that an interaction between ATM monomers and pesticide molecules, or between ATM monomers and metal+pesticides complexes, may contribute to delaying ATM monomers in their nucleo-shuttling. Metals may also delay the ATM nucleoshuttling, but at concentrations higher than 100 µM. Conversely, as already reported, metal salts may cross the nuclear membrane to induce DNA breaks, even at low concentrations [13]. These findings illustrate the fact that any chemical agent may be associated with DNA breaking and DSB recognition inhibition capacities that may be independent. The severity of the response to pesticides or to metal+pesticides complexes may be specifically amplified by an endogenous delay of the ATM nucleo-shuttling, as observed in the RIANS-delayed cells. From these hypotheses, a general mechanistic model can be proposed (Figure 10): metals and/or pesticides that can enter into cells can create, via direct or indirect Fenton-like reactions, an oxidative stress that induces ATM monomerization in cytoplasm and DSB in nucleus (similarly to IR [28] and hydrogen peroxide [63]). Taken together, all the oxidative species, whatever their origin, may result in a proportional number of free ATM monomers in cytoplasm. At certain threshold concentrations, metals and/or pesticides may form some complexes with these ATM monomers, which limits or delays the nucleo-shuttling of the ATM monomers. Hence, taken together, all the ATM-binding elements may result in a proportional number of diffusible ATM monomers. Once in the nucleus, the number of nuclearized ATM monomers may condition the recognition of the DSB specifically induced by the stress. Any unrecognized DSB may lead to toxicity and/or carcinogenicity. In cells already showing an intrinsic delayed RIANS due to the overexpression of some X-proteins (influence of individual and/or tissue factors), the sequestration of ATM monomers in cytoplasm will lead to a more severe response to pesticides and/or metals throughout an additive or supra-additive effect (Figure 10). 

## 5. Conclusions

Together with our previous report published in this journal [13], our findings obtained with six current pesticides of domestic and environmental interest and three independent endpoints (γH2AX, pATM, micronuclei) suggest that exposure to pesticides and/or metals leads to the production of DSB, whether direct or indirect, and involves the ATM protein kinase, a major protein involved in the stress response. As with after an exposure to IR or to metals, exposure to pesticides or to metal+pesticide complexes is consistent with a model based on the production of ATM monomers in cytoplasm that diffuse in the nucleus at a specific rate. The extent of the nucleo-shuttling of ATM depends on the nature of pesticides, their concentration, and the individual factor. At specific concentrations, pesticides complexified with ATM, together with metal or not, prevent the diffusion of ATM monomers in the nucleus, which impairs the DSB recognition and repair, leading to toxicity and/or carcinogenicity. Some specific biomarkers are proposed here to better evaluate the toxicity risks. However, further experiments are needed to better understand and prevent the affinity of ATM to pesticides, and to consolidate this model that may help in the quest of countermeasures of an exposure to pesticide. Furthermore, it will be important to apply a similar approach to commercial preparations that are used in agriculture, and which should contain several additives of which the interplay may impact on biological response.

## 6. Patents

WO2017029450—Individual method predictive of the DNA-breaking genotoxic effects of chemical or biochemical agents.

## Figures and Tables

**Table 1:** Major features of the pesticide molecules used in this study.

Pesticides	Major Chemical Features	Main Use	WHO/IARC Classification
Lindane (HCH)	Organochlorine compound	Agriculture insecticide Treatment against lice and scabies	Moderately acutely toxic Probably carcinogenic in humans (Group 2A)
Atrazine (ATR)	Triazine	Agriculture herbicide	Moderately acutely toxic Not classifiable as to its carcinogenicity to human (Group 3)
Glyphosate (GBH)	Organophosphorus compound	Agriculture systemic herbicide and crop desiccant	GBH toxicity is a subject of controversies Probably carcinogenic in humans (Group 2A)
Permethrin (PER)	Pyrethroid	Agriculture insecticide Treatment against lice and scabies	No evidence for any notable human genotoxicity Not classifiable as to its carcinogenicity to human (Group 3)
Pentachlorophenol (PCP)	Organochlorine compound	Agriculture pesticide Disinfectant	Acute toxicity Carcinogenic to human (Group 1)
Thiabendazole (TBZ)	E233	Agriculture antifungal Antiparasitic	Not yet determined Not classifiable as to its carcinogenicity to human (Group 3)

**Table 2:** Data fit parameters obtained from the pesticides and the metallic species tested.

Metal Species	TMC_>2_ (µM)	Sigmoidal * Data Fit Parameters				
		m1	m2	m3	m4	r
TBZ	0.6	7.94	0.634	0.33 10^1^	0.97	0.98
ATR	1.6	307.63	0.876	9.59 10^7^	0.31	0.99
CuSO_4_	10	43	0	7.07 10^2^	0.75	0.99
CuCl_2_	10	1501	0.13	8.84 10^5^	0.62	0.99
PER	24.3	7.58	0	1.94 10^2^	0.48	0.99
GBH	29.0	39.50	0.105	9.59 10^6^	0.23	0.99
HCH	38.1	295.80	0.447	1.67 10^5^	0.62	0.99
PCP	39.8	4205.60	0.754	0.91 10^2^	1.05	0.98

* The sigmoidal function applied tested for data fitting was y = m1 + (m2 − m1)/(1 + (x/m3)^m4) with m1, m2, m3 and m4 as adjustable parameters. r is the correlation coefficient.

## Data Availability

All the data can be provided on reasonable request.

## Supplementary Materials

The following are available online at https://www.mdpi.com/article/10.3390/biom12020250/s1, Figure S1: Relationship between the TMC_>2_ values of normal- and delayed RIANS cells; Figure S2: Relationship between the TMC_>2_ values from γH2AX and micronuclei data; Figure S3: Relationship between the γH2AX and pATM foci data; Figure S4: Relationship between TMC_>2_, γH2AX/micronuclei ratio and DSB recognition inhibition power with IARC group classification.
