# Supplementary material for: DNA Double-Strand Breaks Induced in Human Cells by 6 Current Pesticides: Intercomparisons and Influence of the ATM Protein
Source: Biomolecules. 2022 Feb 3;12(2):250. doi: 10.3390/biom12020250 (PMC8961571; doi:10.3390/biom12020250)
Supplement: Supplementary file 1 [file biomolecules-12-00250-s001.zip › biomolecules-1558368-supplementary.pdf]

DNA double-strand breaks induced in human cells by 6 current pesticides: intercomparisons and influence of the ATM protein

Supplementary data

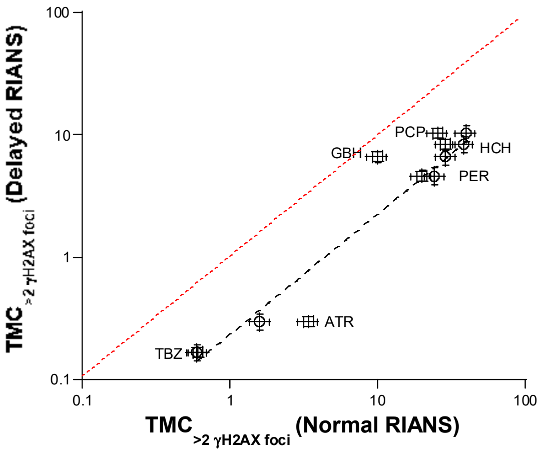

Figure S1: Relationship between the TMC>2 values of normal- and delayed RIANS cells. The means ± standard error (SEM) of the TMC>2 values obtained from the data shown in Fig. 1 with the delayed-RIANS 08HNG cells were plotted against the means ± SEM corresponding TMC>2 values from the normal-RIANS 1BR3 (circles) and 149BR (squares) cells. Data are consistent with the following fitting formula (y=0.238x-0.232; r=0.985). The red dotted line indicates a one-to-one correlation.

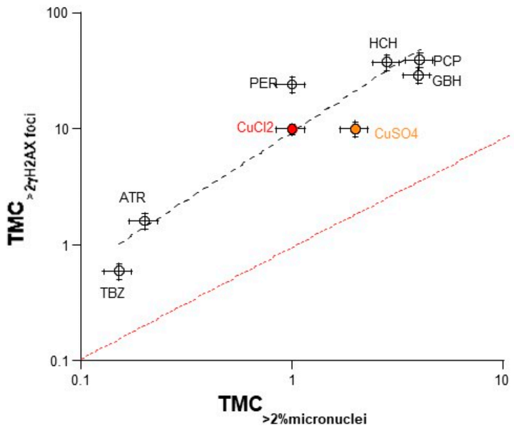

Figure S2: Relationship between the TMC>2 values from γH2AX and micronuclei data. The means of the means ± standard error (SEM) of the TMC>2γH2AX foci values shown in Fig. S1 were obtained from the data shown in Fig. 1 with the RIANS-delayed 08HNG cells were plotted against the means ± SEM of the corresponding TMC>2% micronuclei values obtained from data shown in Fig. 2. the RIANS-normal 1BR3 (circles) and 149BR (squares) cells. Data are consistent with the following fitting formula (y=8.391x+5.18; r=0.875. The red dotted line indicates a one-to-one correlation.

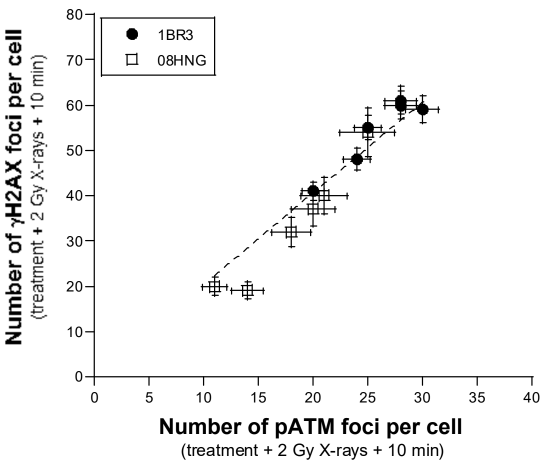

Figure S3: Relationship between the γH2AX and the pATM foci data. The means ± standard error (SEM) of the numbers of γH2AX foci per cell assessed after an incubation for 24 h with the pesticide followed by 2 Gy X-rays and assessed 10 min post-irradiation (data shown in Fig. 4) were plotted against the corresponding means ± SEM of the numbers of pATM foci assessed in the same conditions (data shown in Fig. 6). The dotted line indicates a data fit obeying the following formula: y=2.035x (r=0.875).

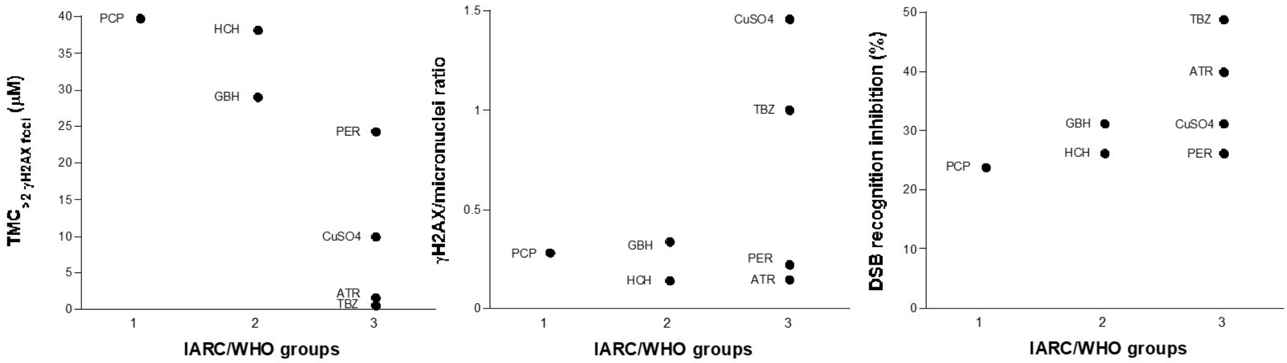

Figure S4: Relationship between TMC>2, γH2AX/micronuclei ratio and DSB recognition inhibition power with IARC group classification. The TMC>2 γH2AX/micronuclei ratio and DSB recognition inhibition power of the different pesticides tested deduced in this study and of $CuSO_4$ deduced in [13] in were plotted against the corresponding IARC groups classification.
